# From Mushrooms to Molecules: Exploring Depsidones in *Ganoderma lucidum* for Antioxidant and Anticancer Applications

**DOI:** 10.3390/molecules30173650

**Published:** 2025-09-08

**Authors:** Sayed H. A. Mohamed, Yehia A.-G. Mahmoud, Mohamed Y. Bediway, Sobhy E. Elsilk, Mohammed Yosri, Kamel Metwally, Nader E. Abo-Dya, Galal Yahya, Mervt Almostafa, Atef A. El-Hela

**Affiliations:** 1 Botany and Microbiology Department, Faculty of Science, Tanta University, Tanta 31527, Egypt; 2 The Regional Center for Mycology and Biotechnology, Al-Azhar University, Nasr City, Cairo 11787, Egypt; 3 Department of Pharmaceutical Chemistry, Faculty of Pharmacy, University of Tabuk, Tabuk 71491, Saudi Arabia; 4 Department of Microbiology and Immunology, Faculty of Pharmacy, Zagazig University, Zagazig 44519, Egypt; 5 Department of Molecular Genetics, Faculty of Biology, Technical University of Kaiserslautern, Paul-Ehrlich Str. 24, 67663 Kaiserslautern, Germany; 6 Department of Chemistry, College of Science, King Faisal University, Al-Ahsa 31982, Saudi Arabia; 7 Pharmacognosy Department, Faculty of Pharmacy, Al-Azhar University, Nasr City, Cairo 11787, Egypt

**Keywords:** *Ganoderma lucidum*, depsidones, antioxidant, LC-MS/MS, anticancer, molecular docking

## Abstract

Fungi are a prolific source of diverse bioactive metabolites, yet many remain unexplored. Among these, depsidones are a rare class of compounds with significant biological potential, but they are seldom reported in mushrooms. This study investigated the medicinal fungus *Ganoderma lucidum*, known for its extensive therapeutic use in traditional medicine. Fruiting bodies were extracted using petroleum ether, ethyl acetate, n-butanol, and methanol. Extracts were screened phytochemically and assessed for total phenolic content and antioxidant activity using the DPPH assay. Ethyl acetate extract exhibited the highest phenolic yield and antioxidant potential and was subsequently evaluated for cytotoxicity against HepG2, HCT116, MCF7, and A549 cancer cell lines. It showed notable anticancer activity with minimal toxicity to normal Vero cells. UHPLC/Q-TOF-MS/MS analysis of *G. lucidum* ethyl acetate extract tentatively identified nine minor depsidones including mollicellin G, simplicildone I, mollicellin B, talaromyone B, simplicildone A, purpactin C, emeguisin B, mollicellin E, and simplicildone D on the basis of high-resolution negative-mode detection and characteristic MS/MS fragmentation patterns. Molecular docking revealed strong binding affinities between these compounds and cancer-related targets (AKT1, CDK2, ERK1, TNFα), with simplicildone D and mollicellin G demonstrating particularly high interactions. These findings provide mechanistic insights into the observed bioactivity and highlight *G. lucidum* as a promising source of therapeutic depsidones for future anticancer drug development.

## 1. Introduction

*Ganoderma lucidum* (*G. lucidum*), is a macrofungus with massive fruiting structures with many beneficial applications [1]. It is thought to promote health and lengthen life [2,3]. This mushroom has long been used in medicinal products and health cures in Asian conventional therapies [4,5]. Researcher interest in medicinal mushrooms has led to the development of useful research on their biologically active elements and their significance [6,7]. Research has shown that *G. lucidum* includes more than 200 distinct kinds of polysaccharides along with more than 400 secondary metabolites [8]. These include triterpenoids (e.g., ganoderic, lucidenic, and ganoderenic acids), other terpenes and sterols, polysaccharides (notably β-D-glucans), phenolic compounds, alkaloids, and peptides, each contributing through distinct and sometimes synergistic mechanisms [9]. With respect to the location of production, the circumstances for development, chemical extraction techniques, and strain selection, the kinds and amounts of biologically active substances in *G. lucidum* can change [10]. The isolation and identification of these metabolites have been accomplished using various separation and purification techniques, such as solvent–solvent partitioning, column chromatography (silica gel, Sephadex LH-20), preparative high-performance liquid chromatography (HPLC), and countercurrent chromatography. Spectroscopic methods, including nuclear magnetic resonance (NMR), Fourier transform infrared spectroscopy (FTIR), ultraviolet–visible spectroscopy (UV–vis), and mass spectrometry (MS), have been pivotal for structure elucidation [11,12,13,14].

Given the extensive use of *Ganoderma* without any reported cases of toxicity, many researchers assert that it is safe and beneficial for consumption [10].

A wide array of distinctive biomolecules derived from natural biological sources and occurring abundantly in nature [15]. Due to their accessibility and affordability, especially in impoverished nations, these metabolites have been used for many years as a basic remedy for a variety of human ailments. Additionally, they are a great source for therapeutic development and research due to their chemical variety and wide range of biological functions [16]. One of the biggest obstacles to the successful use of particular metabolites at the moment is the procedure for the extraction of active molecules [17,18]. Lichens and mushrooms may be a source of phenolic compounds known as depsidones and depsides, which are substances made up of two or three aromatic ring structures of orcinol or β-orcinol connected by an ester bond or an extra ether bond [19].

The variety of structures and diverse biological properties of these compounds have garnered a lot of study attention. There have been reviews of this kind of metabolites in earlier publications. The isolation, structural description, metabolism, and biological activities of many kinds of depsidones identified from fungal sources were covered in many reports [20,21].

Phytochemical constituents and secondary metabolites (over 430) extracted from *G. lucidum* demonstrate a broad range of pharmacological activities, including anticancer, antioxidant, antidiabetic, antihyperlipidemic, antimicrobial, hepatoprotective, cardioprotective, anti-aging, anti-androgenic, antiallergic, analgesic, and anti-frailty effects [9,22]. With well-documented therapeutic potential across diverse disease models, *G. lucidum* is utilized in over 100 commercial formulations, emphasizing its global relevance as a multifunctional natural health product [23].

The purpose of this study is to characterize the phytochemical composition of different solvent extracts of *G. lucidum*, to relate the identified metabolites to their observed biological activities (particularly cytotoxic effects), and to provide mechanistic insights into the relationship between chemical constituents and bioactivity.

We employed liquid chromatography–high resolution tandem mass spectrometry (LC–HRMS/MS) to describe the bioactive chemicals, especially depsidones, which have rarely been reported in G. lucidum, and analyze the biological characteristics of different extracts of *G. lucidum* using different solvents.

To evaluate the anticancer potential of *G. lucidum* extracts, five well-characterized cell lines were selected, each representing a different physiological or pathological context. HepG2 (human hepatocellular carcinoma), HCT116 (human colorectal carcinoma), MCF7 (human breast adenocarcinoma), and A549 (human lung adenocarcinoma) are widely used in cytotoxicity studies due to their relevance in prevalent cancer types and their distinct molecular and metabolic profiles. Vero cells (African green monkey kidney epithelial cells) were included as a non-cancerous normal cell model to assess selectivity and potential cytotoxicity towards healthy cells.

Molecular docking provides valuable insights into the molecular basis of the observed cytotoxic activity and supports the pharmacological potential of these naturally derived compounds. In this study, molecular docking was employed to evaluate the binding affinity and interaction profiles of the bioactive constituents identified in the ethyl acetate extract of *G. lucidum*. Specifically, nine depsidone compounds were subjected to in silico docking against key cancer-related protein targets, including AKT1, CDK2, ERK1, and TNFα. This computational approach aimed to predict the potential anticancer mechanisms of these molecules by analyzing their binding modes, interaction energies, and key stabilizing forces.

## 2. Results and Discussion

### 2.1. Qualitative Phytochemical Screening and Antioxidant Potential

Qualitative phytochemical screening for *G. lucidum* upon extraction using various solvents can be seen in Supplementary Table S1. Sterols and/or triterpenes could be detected upon extraction using petroleum ether. However, anthraquinone derivatives and/or depsidones were the most predominant group detected upon using ethyl acetate for extraction. Moreover, none of the qualitative applied tests resulted in a positive outcome upon extraction using n-butanol for extraction. Finally, using methanol for extraction resulted in the production of many chemical groups, including carbohydrates, flavonoïdes, tannins, and alkaloids. The nutritive benefits of *G. lucidum* mushrooms have been the subject of various investigations [24]. According to Ahmad et al. [25], studies on the biochemical makeup of this mushroom have identified a number of categories of chemicals with biological functions that promote health.

In this work, various solvents were applied to extract *G. lucidum* in accordance with earlier studies [26], by which the bioactive components of mushrooms can be extracted using polar or non-polar solvents. A methanol-based extract of newly harvested fruiting structures from *G. lucidum* inhibits the development of several microorganisms, according to Mondal et al. [27].

Quantitative analysis for phenolic compounds in *G. lucidum* reflected that application of ethyl acetate in extraction resulted in highest yield of phenolic compounds, with a level of 9.87 ± 0.71 mg/g. However, using methanol in extraction resulted in a moderate yield of phenolic compounds with a level of 4.27 ± 0.11 mg/g. Finally, using either petroleum ether or n-butanol resulted in a weak yield of phenolic molecules (Table 1). Several phenolic acids found in *G. lucidum* that have antioxidant action were the subject of further evidence reported by numerous researchers [28,29]. Rahman et al. examined ascorbic acid and flavonoid components in depth in their 2020 study [30].

**Figure 1:**
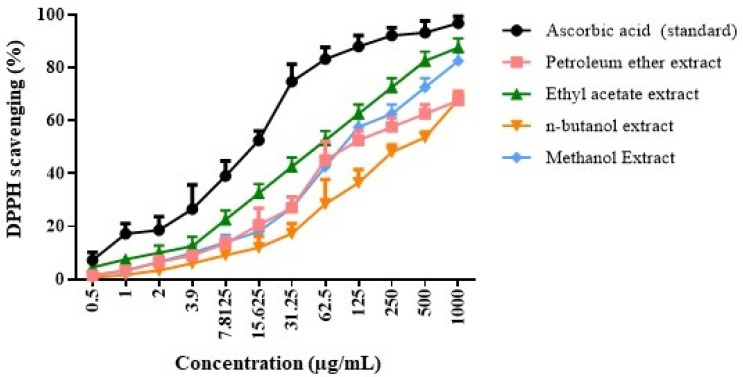
DPPH assay for *G. lucidum* extract upon using various solvents including petroleum ether, ethyl acetate, n- butanol, and methanol versus the ascorbic acid standard (data are presented as means ± SD).

The results of the antioxidant impact of various extracts of *G. lucidum* can be seen in Figure 1. It can be noticed that the ethyl acetate extract had the highest antioxidant impact with IC_50_ = 39.87 ± 1.7 µg/mL, while the antioxidant action of petroleum ether, n-butanol, and methanol had IC_50_ values of = 85.87 ± 0.8, 165.17 ± 1.2, and 55.47 ± 0.71 µg/mL, respectively. Ascorbic acid had a level of IC_50_ = 10.21 ± 0.77 µg/mL. The antioxidant activity of *G. lucidum* extracts is largely attributed to their rich phytochemical content, including triterpenoids, polysaccharides, polyphenols, flavonoids, and ascorbic acid, which can scavenge free radicals and protect against oxidative stress [31,32].

### 2.2. Cytotoxicity Against Cancer Cell Lines

**Figure 2:**
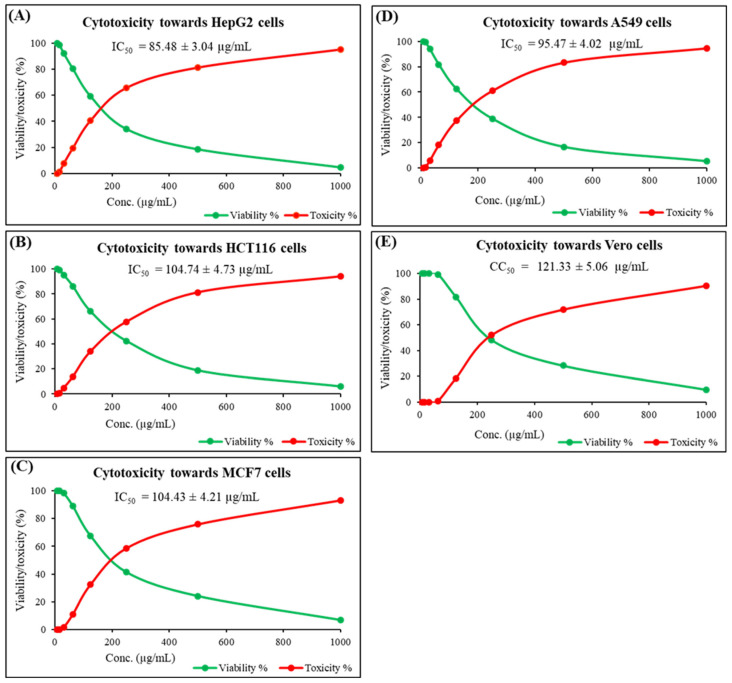
Cytotoxicity impact of *G. lucidum* ethyl acetate extract on (**A**) HepG2 cells, (**B**) HCT116 cells, (**C**) MCF7 cells, (**D**) A549 cells, and (**E**) Vero cells. Data are represented as means ± SD.

*G. lucidum* ethyl acetate extract was tested against various cancer cells as well as a normal cell line, as depicted in Figure 2A–E. This combination of cell lines provides a robust platform to evaluate both the anticancer efficacy and safety profile of the tested extracts. It could be noticed that the tested extract had promising antitumor towards hepatocellular carcinoma cells (HepG2), colon carcinoma cells (HCT116), breast carcinoma cells (MCF7), and lung carcinoma cells (A549), with IC_50_ values of =85.49 ± 3.04, 104.74 ± 4.73, 104.43 ± 4.21, and 95.47 ± 4.02 µg/mL, respectively. Besides, it has a potent impact on mammalian cells from African green monkey kidney (Vero), representing normal cells, with CC_50_ = 121.33 ± 5.06 µg/mL.

It is anticipated that the discovery of novel drugs to treat human malignancies would make use of mushroom extracts [33]. The present findings indicate that *G. lucidum* extracts exert varying degrees of cytotoxicity against multiple human cancer cell lines, with notable selectivity when compared with non-cancerous Vero cells. This aligns with a growing body of evidence supporting the anticancer potential of medicinal mushrooms [34,35]. Beyond their historical role in traditional medicine, numerous fungal species have yielded pharmacologically important compounds, including clinically used anticancer agents such as taxanes and bisindole alkaloids [36,37].

In the case of *G. lucidum*, several classes of bioactive metabolites are implicated in its cytotoxic effects. Polysaccharides, particularly β-D-glucans, have been widely reported to modulate immune responses, enhance host defense mechanisms, and inhibit tumor growth both directly and indirectly [38]. Triterpenoids, such as ganoderic and lucidenic acids, have demonstrated the ability to induce apoptosis, cause cell cycle arrest, and suppress metastatic potential in various cancer cell models. Phenolic compounds and sterols present in *G. lucidum* may further contribute by exerting antioxidant and pro-oxidant effects that disrupt cancer cell survival pathways.

The differential sensitivity observed among the different cancer cells may reflect differences in their genetic background, receptor expression, and metabolic profiles, suggesting that specific phytochemicals in *G. lucidum* could target multiple molecular pathways in a tumor-type-dependent manner. Importantly, the relatively low cytotoxicity toward Vero cells highlights the potential therapeutic window and selective cytotoxicity of the extracts.

The cytotoxic effects of *G. lucidum* ethyl acetate extract observed in this study can be directly linked to the classes of metabolites identified during phytochemical screening. The ethyl acetate extract is rich in polar bioactivities, including carbohydrates/glycosides, flavonoids, tannins/phenolics, alkaloids, and triterpenes. These compound classes have well-documented anticancer mechanisms. For example, polysaccharides such as β-D-glucans can stimulate immune-mediated tumor suppression and induce apoptosis, while phenolics and flavonoids may inhibit cancer cell proliferation through antioxidant, pro-oxidant, and signaling pathway modulation [39,40,41].

It is worth mentioning that the n-butanol and petroleum ether extracts were largely devoid of the key anticancer-associated metabolites identified in ethyl acetate fraction. 

The low toxicity of ethyl acetate extract toward Vero cells suggests that these phytochemicals may selectively target cancer cell pathways without significantly affecting normal cells’ viability.

These observations underscore the direct relationship between the phytochemical profile of each extract and its bioactivity, supporting the hypothesis that the anticancer potential of *G. lucidum* is mediated through a synergistic interplay among polysaccharides, triterpenoids, phenolic compounds, and flavonoids.

Taken together, these findings reinforce the promise of *G. lucidum* as a source of leading compounds for anticancer drug development, warranting further mechanistic and in vivo studies.

### 2.3. UHPLC/Q-TOF-MS-MS Analysis

**Figure 3:**
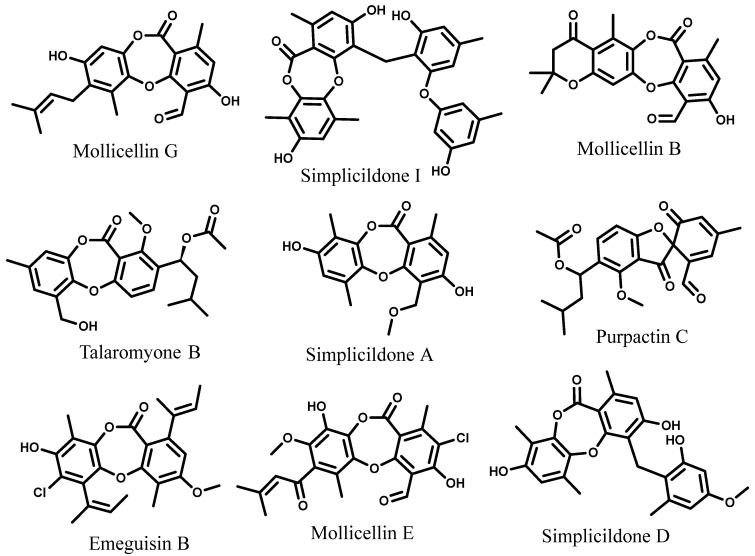
Names and structures of depsidones detected in the ethyl acetate extract of *G. lucidum*.

UHPLC/Q-TOF-MS-MS analysis for ethyl acetate extract of *G. lucidum* reflected the existence of nine different minor compounds, which have been tentatively identified as depsidones using a high-resolution detection (negative mode) in order to determine their molecular formula identification on the basis of their common fragmentation ions in the MS/MS spectra (Figure 3). These molecules were mollicellin G (C_22_H_23_O_7_**)** [37], simplicildone I (C_31_H_28_O_8_) [38], mollicellin B (C_21_H_18_O_7_) [42], talaromyone B (C_23_H_26_O_7_) [43], simplicildone A (C_18_H_18_O_6_) [44], purpactin C (C_23_H_21_O_7_) [45], emeguisin B (C_24_H_25_ClO_5_) [46], mollicellin E (C_22_H_19_ClO_8_) [37], and simplicildone D (C_25_H_24_O_7_) [38].

According to the LC-MS studies, the ethyl acetate extract of *G. lucidum* included nine peaks of depsidones. These peaks were accurately recognized by mass measurement, and the molecular structures were subsequently tentatively characterized using MS/MS fragmentation patterns (Supplementary Figure S1 and Table 2). This is the first work to characterize and provisionally identify these groups of compounds in *G. lucidum.* The results of our study are in line with the findings of an investigation that found that naturally produced depsidones have outstanding biological properties. Given the rapid advancements in studies, the search for novel active depsidones from natural origins may prove to be an effective method for developing new herbal remedies [47].

Depsidone fragmentation ions are produced via cyclization after the straightforward loss of CO_2_, H_2_O, and CH_2_O. A common deprotonated molecule was found in all depsidones, along with a fragment that showed an impairment of 44 mass units. It is assumed that this neutral CO_2_ loss on the depsidone scaffold’s central core provides a dibenzofuran moiety [47]. The order in which these compounds elute further supports the structural designations of these depsidones [48,49].

### 2.4. Virtual Screening and Protein–Ligand Interactions

**Figure 4:**
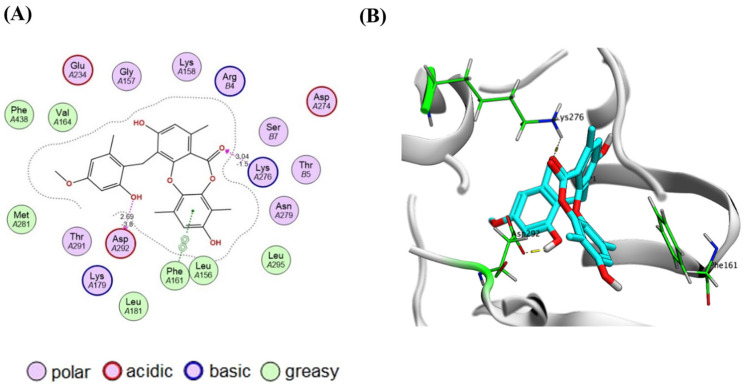
Binding interactions of simplicildone D with AKT1 (PDB code: 3OCB). (**A**) Two-dimensional binding interactions; (**B**) 3D pose (ligand atoms in cyan and critical amino acid residues in green).

Docking analysis indicated that simplicildone D displayed the highest binding affinity (docking score = −7.77 kcal/mol, RMSD = 1.68 Å) to AKT1 (PDB code: 3OCB, *Homo sapiens*) [50]. Inspection of the docking poses revealed two key hydrogen bonding interactions that contribute to binding stabilization. The phenolic hydroxy group of the benzyl moiety donates a hydrogen bond (E = −3.8 kcal/mol, distance = 2.69 Å) to the side chain carboxylate group of Asp292 which is a constituent part of the DFG motif. In addition, the lactone carbonyl group accepts a hydrogen bond (E = −1.5 kcal/mol, distance = 3.04 Å) from the side chain amino group of Lys276 located in the catalytic loop of AKT1. On the other hand, the xylenol ring π-π stacks the phenyl side chain of Phe161 located within the G-loop of the kinase domain (Figure 4). It appears that the free rotation offered by the benzyl moiety enables the correct positioning of the hydroxyl group near Asp292 for a strong hydrogen bonding interaction. In simplicildone I, hydrogen bond formation may be sterically hindered by the bulky phenoxy group attached to the benzyl moiety. In other compounds, a phenolic hydroxy group is either absent or directly attached to a rigid tricyclic ring structure, which minimizes the binding flexibility.

**Figure 5:**
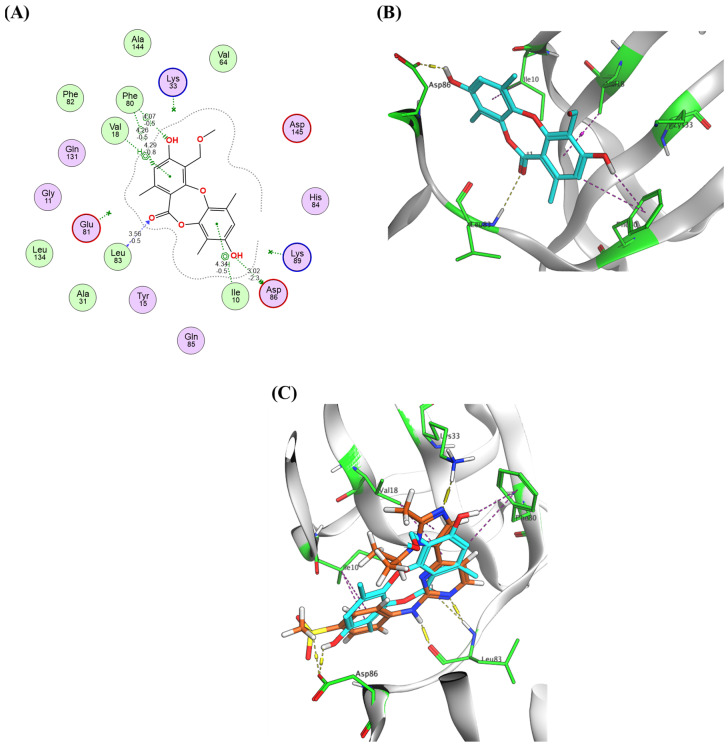
Binding interactions of simplicildone A with CDK2 (PDB code: 6GUE). (**A**) Two-dimensional binding interactions; (**B**) 3D pose (ligand atoms in cyan and critical amino acid residues in green); (**C**) overlay of Compound 5 with the co-crystallized ligand inside the active site.

The docking results of the target compounds with CDK2 (PDB code: 6GUE, *Homo sapiens*) [51] showed that simplicildone A exhibited the highest binding affinity (docking score = −7.72 kcal/mol, RMSD = 1.75 Å) with crucial interactions with the CDK2 hinge residues Asp86 and Leu83. The xylenol hydroxy group donated a hydrogen bond to the side chain carboxylate group of Asp86, and the lactone carbonyl oxygen accepted a hydrogen bond from the backbone NH group of Leu83. It was also observed that Compound 5 establishes several hydrophobic contacts such as the π-H interactions with Ile10 and Val18 located the ATP-binding pocket in the N-terminal lobe of the kinase domain and an H-π interaction with Phe80 located near the hinge region of AKT1, thereby contributing to binding stabilization (Figure 5). Notably, the relatively small molecular size of simplicildone A compared with the other compounds examined indicates that steric parameters may play a role in binding to hotspots of CDK2. This corresponds with previous findings that utilized contour maps to demonstrate the significance of electrostatic, hydrophobic, hydrogen bond donor, and steric fields in CDK2 binding [52].

**Figure 6:**
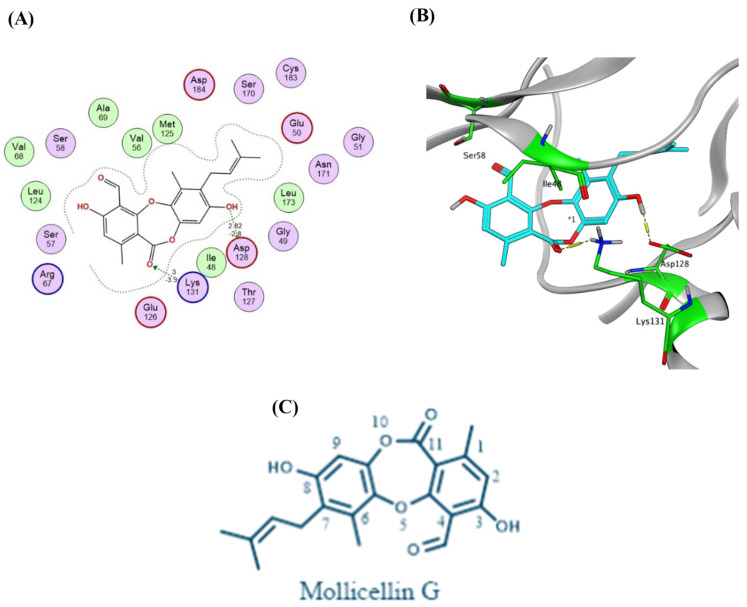
Binding interactions of mollicellin G with ERK1 (PDB code: 2ZOQ). (**A**) Two-dimensional binding interactions; (**B**) 3D pose (ligand atoms in cyan and critical amino acid residues in green). (**C**) Chemical structure of mollicellin G, showing the numbering of carbon atoms.

Inspection of the docking poses of the target compounds with ERK1 (PDB code: 2ZOQ, *Homo sapiens*) [53] revealed that mollicellin G displayed the highest binding affinity (docking score = −7.84 kcal/mol, RMSD = 1.78 Å) establishing two hydrogen bonding interactions. The hydroxy group adjacent to the alkylidene moiety donates a hydrogen bond (E = −2.80 kcal/mol, distance = 2.88 Å) to the carboxylate side chain of Asp128, which is a part of the HRD motif of ERK1. Additionally, the lactone carbonyl group accepts a strong hydrogen bond (E = −3.90 kcal/mol, distance = 3.00 Å) from the side chain amino group of Lys131 in the catalytic loop of the kinase domain (Figure 6A,B). The pentylidene moiety of mollicellin G may influence its conformation in a way that positions the phenolic hydroxyl and lactone carbonyl groups to form strong hydrogen bonds. While mollicellin G; simplicildones A, D, and I; and emeguisin B have a C-8 phenolic hydroxyl group and a carbonyl group at C-11, only mollicellin G can form hydrogen bond interactions with Asp128 and Lys131 of ERK1. This is probably because mollicellin G does not have a C-9 methyl group, which could cause steric hindrance that might interfere with these interactions in the other molecules (Figure 6C).

**Figure 7:**
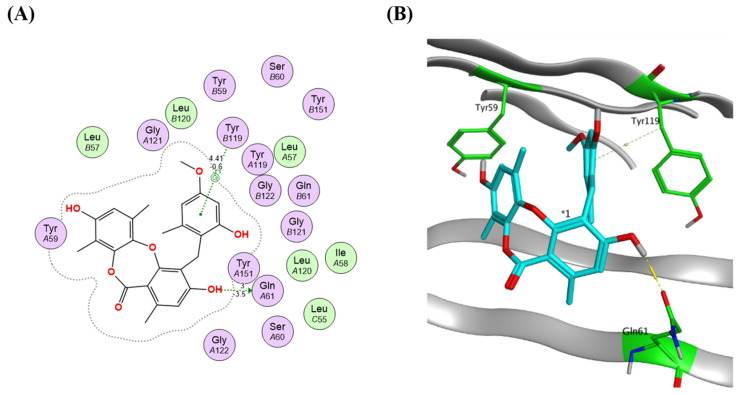
Binding interactions of simplicildone D with TNFα (PDB code: 2AZ5). (**A**) Two-dimensional binding interactions; (**B**) 3D pose (ligand atoms in cyan and critical amino acid residues in green).

Simplicildone D was found to display the highest binding affinity (docking score = −7.21 kcal/mol, RMSD = 0.79 Å) to TNFα (PDB code: 2AZ5, *Homo sapiens*) [54]. A key hydrogen bonding interaction is established between the hydroxy group ortho to the benzyl moiety and the side chain carbonyl group of Gln61 located within a β-strand of the “jelly roll” β-sandwich strand. On the other hand, a pi-H interaction is established between the phenyl ring of the benzyl moiety and the methylene group of Tyr119 located in the receptor-binding interface of the TNF-α homotrimer (Figure 7). This pi-H interaction displayed by the benzyl moiety may play a role in stabilizing the hydrogen bonding interaction established by the adjacent hydroxyl group.

## 3. Materials and Methods

### 3.1. Acquisition of Mushrooms

*G. lucidum* fresh fruiting bodies (FFBs) were acquired from the Egyptian Agriculture Research Center in Giza, Egypt. The fungus was classified using established norms and by comparing them to voucher samples (Voucher ID: 0245) from the Tanta University Herbarium’s Microbiology Department (Tanta, Egypt) [55]. Before being kept in a dehydrator at −20 °C until further research, the FFBs were cleaned with deionized water, dried, chopped into pieces (approximately 1.0 cm^2^), and lyophilized using a FreeZone 2.5 Liter Laboratory Freezing Dry System (Kansas City, MA, USA) [56].

### 3.2. Production of the Mushroom Extracts

The stored fruiting bodies were extracted using various solvents (petroleum ether, ethyl acetate, n-butanol or methanol) at 10% (*w*/*v*) in four separate containers. Each specimen was pulverized using a laboratory mixer (Fisher Scientific, Waltham, MA, USA) and then refrigerated for the whole night. An ultrasonicator (Fisher Model 200, New York, NY, USA) was used to blast down these mushroom cells for 30 min at 30 kHz. After centrifugation of the specimens for 20 min at 10,000× *g* to exclude debris, the resulting supernatants were obtained in a separator (Beckman, Brea, CA, USA). Each of the specimens were stored at −20 °C, and the extracts were concentrated by drying by air [57].

### 3.3. Qualitative Screening of Phytochemicals and Total Phenolics in Various Prepared Extracts

The phytochemicals were screened qualitatively in accordance with Nortjie et al. [58]. The total amount of phenolic content was estimated in various samples through mixing 3 mL (70 g/L) of sodium carbonate, 2.0 mL of Folin–Ciocalteu solution, and 100 μL of various extracts. After 20 s of shaking, containers were stored at 37 °C for an hour. At 750 nm, absorbance was determined with a Shimadzu 310 spectrometer (Kyoto, Japan) [59].

### 3.4. Antioxidant Assay of Different Extracts

In order to assess the evaluated samples’ antioxidant ability, a solution of ethanol with 0.1 mM DPPH was used. The produced solution (1 mL) was mixed with 3 mL of different specimens in ethanol (3.9−1000 µg/mL). Following a vigorous shake, the mixture was allowed to stand for 35 min at room temperature. Absorbance was assessed at 520 nm [60].

### 3.5. Cytotoxicity Examination

HepG2 (human hepatocellular carcinoma), HCT116 (human colorectal carcinoma), MCF7 (human breast adenocarcinoma), A549 (human lung adenocarcinoma), and Vero (African green monkey kidney epithelial cells) cell lines were used to evaluate the samples’ in vitro cytotoxic impact employing the 3-(4,5-dimethylthiazol-2-yl)-2,5-diphenyl tetrazolium bromide (MTT) test. The American Type Culture VACSERA Company provided the cells. After that, 96-well plates were filled with 1.1 × 10^5^ cells/mL, and they were incubated for 24 h at 37 °C with 5% CO_2_. After changing the culture medium, six distinct concentrations (1000–32.25 µg/mL) of each extract dissolved in DMSO (dimethyl sulfoxide) were added (the final conc. of DMSO was kept below 0.5% *v*/*v*), and the mixture was cultured for 24 h. The culture media were then taken out and cleaned using phosphate-buffered saline (PBS). As explained for the cytotoxicity experiment, the absorbance measurements of formazan crystals utilized in the MTT reagent were used to assess the life span of treated cells and cells that were not treated. Lastly, a microplate reader was used to determine the absorbance at 570 nm [61].

### 3.6. UHPLC/Q-TOF-MS-MS Testing

Applying the Micromass, Quattro Micro TM (Waters, Milford, MA) technology, high-resolution MS/MS analysis was carried out. After dissolving 50 mg of the sample in 1 mL of the recovered solvent (water–methanol–acetonitrile, 50:25:25 *v*/*v*), the mixture was vortexed for 2 min and then ultrasonically sonicated for 10 min. Next, 50 µL of the stock liquid was diluted with 1000 µL of the reconstitution solvent and centrifuged for 10 min at 10,000 rpm. In the end, 2.5 µg/µL was the injected quantity, and 10 µL was injected in negative mode. We used a mobile phase gradient elution (A, negative mode) and 10 µL of the reconstitution solvent as a blank sample. An ammonium formate buffer (pH 8.5) with 1% methanol and a 0.3 mL flow rate on a column (2.1 × 150 mm, 5 µm) was used for the mobile phase C with 100% acetonitrile at 40 °C [62].

### 3.7. Molecular Docking and Protein–Ligand Interactions

Molecular docking investigations were carried out using the Molecular Operating Environment (MOE) 2024.06 software suite (Chemical Computing Group Inc., Montreal, QC, Canada) [63]. The simulations were run on a Windows 11 workstation with an Intel^®^ Core™ i5 processor, 16 GB RAM, and an NVIDIA RTX GPU. Protein preparation was carried out using the MOE QuickPrep module, which included the addition of hydrogen atoms, the correction of protonation states at physiological pH (7.4), and the allocation of partial charges using the Amber10: EHT forcefield. All ligands, including both the reference compounds and the identified extract components, were produced for molecular modeling using the MOE sketch from their chemical names, which were obtained from the PubChem and Zinc databases. The ligands were prepared using 3D protonation, energy minimization with the MMFF94x forcefield, and partial charge assignment (MMFF94x) along with geometry optimization. The Dock module in the Molecular Operating Environment (MOE) program was used to run molecular docking simulations. The docking methodology utilized the Triangle Matcher method to place the ligands at first, then the London dG function to score them again and finally the GBVI/WSA dG scoring function for refinement. A forcefield-based refinement was used in the binding site and kept the top 10 poses for each ligand for more study. To make sure that the docking method was reliable, the co-crystallized ligand was redocked into its known binding site. The experimental and predicted poses must have a root mean square deviation (RMSD) of ≤2.0 Å for the validation to be successful [63].

Protein–ligand interactions were visualized and analyzed using MOE’s LigX tool, which identified key hydrogen bonds, hydrophobic contacts, salt bridges, and π–π or cation–π interactions. Binding affinity was estimated from the docking score (S score, kcal/mol) [63].

### 3.8. Statistical Evaluation

The results are expressed as the mean ±SD, and each experiment was run three times. The results were tested using Graph Pad Prism V8 (CA, USA) software, and the Tukey test was used to examine the means before one-way testing of variations (one-way ANOVA). The examination was deemed significant when the *p*-value was less than 0.05.

## 4. Conclusions

The present study highlights the value of *G. lucidum* as a rich source of bioactive compounds, particularly when extracted using ethyl acetate. This solvent not only yielded the highest concentration of phenolic compounds but also demonstrated superior antioxidant and substantial cytotoxic activities. Notably, LC-MS analysis revealed nine unique depsidone derivatives, provisionally identified for the first time in *G. lucidum*. Molecular docking further substantiated their therapeutic relevance, showing high binding affinity to key cancer-related targets like AKT1, CDK2, ERK1, and TNFα. These interactions suggest the potential of these natural compounds in future anticancer drug development. The study reinforces the importance of solvent selection in phytochemical extraction and provides a foundation for future investigations into the pharmacological utility of depsidones. Overall, the findings support the promising role of *G. lucidum* in nutraceutical and pharmaceutical applications, particularly as a source of natural depsidone-based therapeutics.

## Figures and Tables

**Table 1:** Total phenolics of all extracts of *G. lucidum* (data are represented as means ± SD).

Sample Code	(1) Petroleum Ether Extract	(2) Ethyl Acetate Extract	(3) N-Butanol Extract	(4) Methanol Extract
**Total Phenolics (mg/g)**	0.21 ± 0.12	9.87 ± 0.71 *	0.11 ± 0.01	4.27 ± 0.11

* *p* ≤ 0.05 (using one-way ANOVA followed by a post hoc test).

**Table 2:** The result of depsidones tentatively identified by UHPLC/Q-TOF-MS-MS analysis (negative mode) in the ethyl acetate extract of *G. lucidum*.

No	Name of the Depsidone Compound	Rt (Min)	Mol. Weight [M-H]-	Error (ppm)	Characteristic Frag.	References
1	Mollicellin G C_22_H_23_O_7_	1.11235	398.1352	3.5	202.037, 238.0756, 396.855, 98.128450, 188.0453.195.0500, 200.0599, 218.0507, 218.105, 278.0984, 308.097, 330.107, 362.144, 398.10425	[37]
2	Simplicildone I C_31_H_28_O_8_	1.21	527.1707	1	527.1746, 376.212, 348.402, 177.542	[38]
3	Mollicellin B C_21_H_18_O_7_	1.244983	381.0977	0.8	381.097, 366.371, 322.804, 179.374, 167.529, 217.1236, 219.04, 219.096, 243.076, 261.0647, 363.1362, 381.102	[42]
4	Talaromyone B C_23_H_26_O_7_	1.31	413.4565	−8.4	413.1656, 353.221, 339.391, 283.024	[38]
5	Simplicildone A C_18_H_18_O_6_	1.3228	329.103	1.5	329.103, 314.453, 314.409, 85.1782, 285.324, 155.164, 154. 328, 91.443, 76.221	[44]
6	Purpactin C C_23_H_21_O_7_	1.43	408.1255	4.6	408.125, 393.851, 365.344	[45]
7	Emeguisin B C_24_H_25_ClO_5_	1.72	427.8752	−13.1	112.986, 247.071, 256.93, 265.130, 293.103, 337.1281, 427.167, 381.2082, 71.427,1349, 247.0716, 398.021, 427.1356	[46]
8	Mollicellin E C_22_H_19_ClO_8_	3.390283	445.0675	−3.4	445.0675, 312.865, 220.674, 196.034, 191.350, 225.0928, 237.0142, 250.097, 264.9148, 265.108, 279.008, 279.034, 293.046, 309.069, 313.110 361.118, 377.101, 386.1417, 445.069	[37]
9	Simplicildone D C_25_H_24_O_7_	7.60215	435.1465	4.8	435.1465, 429.432, 381.246, 201.176, 154.369, 91.66, 76.33	[38]

## Data Availability

All results included in this work are available with the corresponding author on reasonable request.

## Supplementary Materials

The following supporting information can be downloaded at: https://www.mdpi.com/article/10.3390/molecules30173650/s1; Qualitative phytochemical screening of *G. lucidum* extracts (Supplementary Table S1) and MS/MS fragmentation patterns of *G. lucidum* ethyl acetate extract (Supplementary Figure S1).

## Abbreviations

The following abbreviations are used in this manuscript.

*G. lucidum*	*Ganoderma lucidum*
FFB	Fresh fruiting bodies
LC-MS/MS	Liquid chromatography–mass spectrometry/mass spectrometry
UHPLC/Q-TOF-MS-MS	Ultra-high-performance liquid chromatography/quadrupole time-of-flight mass spectrometry
MTT	3-(4,5-Dimethylthiazol-2-yl)-2,5-diphenyltetrazolium bromide
DPPH	2,2-diphenyl-1-picrylhydrazyl
PBS	Phosphate-buffered saline
MOE	Molecular Operating Environment
RMSD	Root mean square deviation
SD	Standard deviation
ANOVA	Analysis of variance
AKT1	RAC-alpha serine/threonine-protein kinase 1
CDK2	Cyclin-dependent kinase 2
ERK1	Extracellular signal-regulated kinase 1
TNFα	Tumor necrosis factor alpha
Vero	African green monkey kidney cells (normal cell line)
IC_50_	Half maximal inhibitory concentration
CC_50_	50% cytotoxic concentration
PDB	Protein Data Bank
