# Supplementary material for: From Mushrooms to Molecules: Exploring Depsidones in Ganoderma lucidum for Antioxidant and Anticancer Applications
Source: Molecules. 2025 Sep 8;30(17):3650. doi: 10.3390/molecules30173650 (PMC12430494; doi:10.3390/molecules30173650)
Supplement: Supplementary file 1 [file molecules-30-03650-s001.zip › molecules-3804905-supplementary.pdf]

Supplementary Table 1. Preliminary qualitative phytochemical screening of *G. lucidum* extracts

Test for	Petroleum ether extract	Ethyl acetate extract	n-butanol extract	Methanol Extract
**Crystalline sublimate**	-ve	-ve	-ve	-ve
**Volatile oil**	-ve	-ve	-ve	-ve
**Carbohydrates and/or glycosides**	-ve	+ve	-ve	+ve
**Flavonoïdes : Aglycons**	-ve	-ve	-ve	+ve
**Glycosides**	-ve	-ve	-ve	+ve
**Tannins and or phenolic compounds**	-ve	+ve	-ve	+ve
**Sterols and/or triterpenes**	++ve	+ve	-ve	-ve
**Saponines**	-ve	-ve	-ve	-ve
**Cardiac glycosides**	-ve	-ve	-ve	-ve
**Alkaloids and or nitrogenous compounds**	+ve	-ve	-ve	+ve
**Anthraquinone derivatives and or depsidones**	-ve	++ve	-ve	-ve
**Oxidase enzyme**	-ve	-ve	-ve	-ve

–ve Negative result: The test did not detect that constituent in the extract (below detection limit).
+ve Positive result (low to moderate presence): The test detected the constituent in a noticeable but not strong amount.
++ve Strong positive result: The test detected the constituent in a high concentration

IDA Survey from IDA-NEG-221228-SM0208-4.wiff (sample 1) - IDA-NEG-221228-SM0208-4

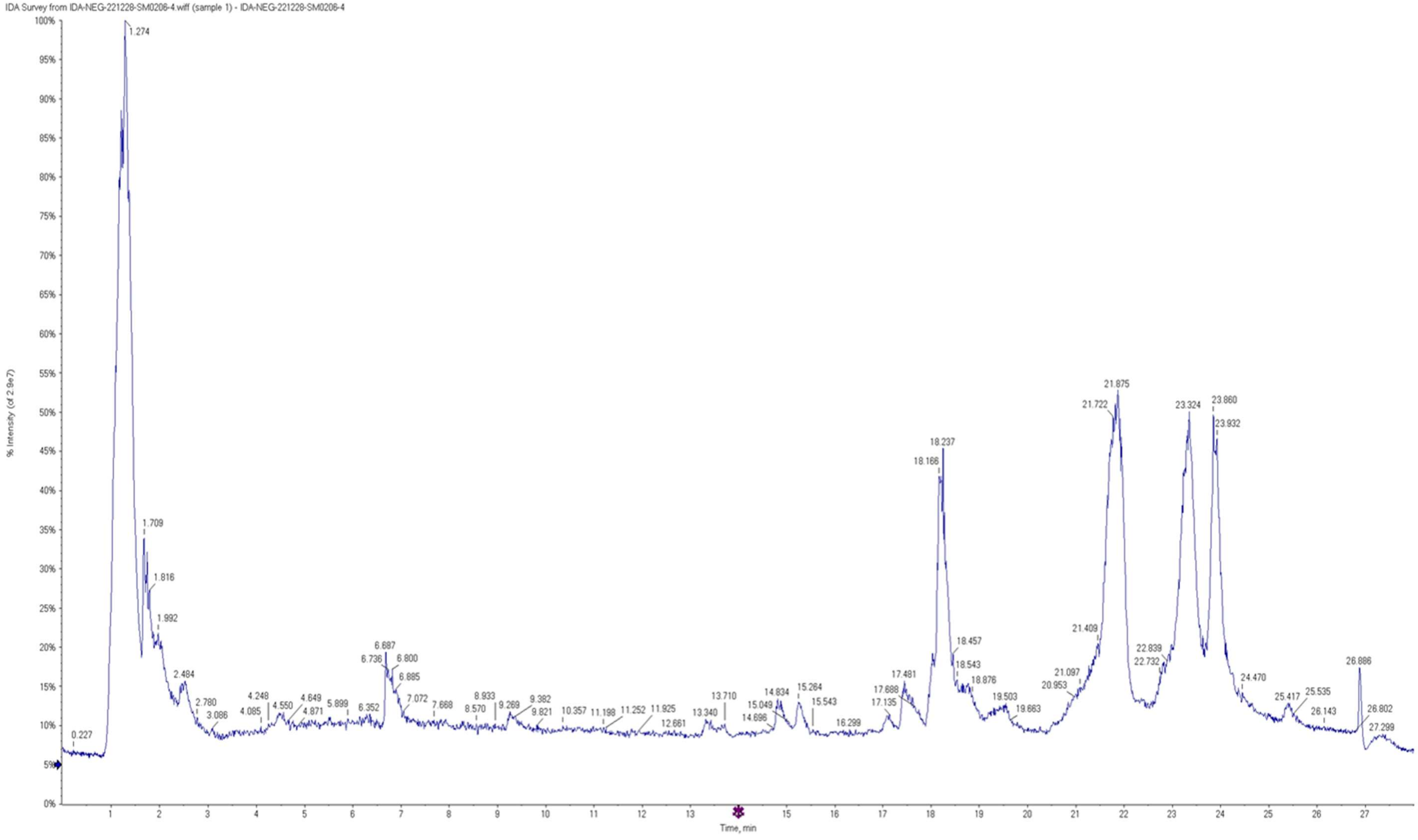

Supplementary Figure S1. UHPLC/Q-TOF-MS-MS (Negative mode) for analysis of in ethyl acetate extract of G. lucidum
